# On the Epidemiology of Xenoma-Forming Microsporidia in Wild Caught Fish from Macaronesia (FAO34)

**DOI:** 10.3390/vetsci12121121

**Published:** 2025-11-26

**Authors:** Kevin M. Santana-Hernández, Eva Betancor, Ana S. Ramírez, Begoña Acosta, Miriam Rodríguez, Emilio Soler-Onís, José Pestano, Eligia Rodríguez-Ponce

**Affiliations:** 1 Department of Pathology, Faculty of Veterinary Science, Universidad de Las Palmas de Gran Canaria (ULPGC), 35413 Las Palmas, Spain; kevin.santana106@alu.ulpgc.es (K.M.S.-H.); bego.acosta@ulpgc.es (B.A.); miriam.rodriguez@ulpgc.es (M.R.); eligia.rodriguezponce@ulpgc.es (E.R.-P.); 2 Genetic Laboratory, Faculty of Medicine, ULPGC, 35016 Las Palmas, Spain; eva.betancor@ulpgc.es (E.B.); jose.pestano@ulpgc.es (J.P.); 3 Observatorio Canario de HABs, FCPCT-ULPGC, 35214 Las Palmas, Spain; esoler@marinebiotechnology.org

**Keywords:** commercial fish, FAO 34, *Glugea*, microsporidia, parasite, xenomas

## Abstract

Microsporidia are a group of obligate intracellular parasites with a broad host range. These parasites can cause mild to severe harm to their hosts, making them important targets for research. However, studies on common wild-caught fish from the Atlantic Eastern Central Zone (FAO Area 34) remain scarce. 576 individuals across 35 fish species from this region were collected from local markets between 2011 and 2019. The specimens were dissected, and lesions were examined using histology, wet mounts, PCR, and transmission electron microscopy. However, microsporidian infection was detected only in round sardinella (*Sardinella* (*S.*) *aurita*), with 19 out of 39 individuals infected. Parasitized fish weighed nearly half as much as non-infected ones, indicating a potentially significant economic impact. Molecular and ultrastructural analyses identified the parasite as *Glugea* (*G.*) *thunni*, a species previously reported only from the Mediterranean Sea. This study provides the first record of *G. thunni* in FAO Area 34, with round sardinella likely serving as its primary host. This and other closely related sequences may represent a single taxonomic entity or, more likely, a complex of a few cryptic species geographically separated across the Mediterranean Sea and the eastern and western North Atlantic regions.

## Simple Summary

Microsporidia are intracellular parasites that can infect many animal species, but little is known about their presence in wild fish from the Atlantic Eastern Central Zone (FAO Area 34). Between 2011 and 2019, 576 fish from 35 species were examined using several laboratory methods. Microsporidian infection was detected only in round sardinella with 19 of 39 individuals infected. Infected fish weighed about half as much as healthy ones, suggesting potential economic losses. Molecular and microscopic analyses identified the parasite as a *Glugea* species previously known only from tunas in the Mediterranean Sea. This study reports its presence for the first time in FAO Area 34, with round sardinella as its likely primary host.

## 1. Introduction

The phylum Microsporidia (Fungi: Opisthosporidia) is considered to hold over 1600 species in approximately 200 genera [1,2,3]. These organisms are obligate intracellular parasites with much importance in human and veterinary medicine [4]. Many of them are host-specific, while others may cause disease in a variety of hosts or even represent a zoonotic risk [5,6,7].

More than 160 microsporidians have been described in fish [1,2,3] and some of them are reported to cause infections in those harvested from Eastern Central Atlantic region (FAO 34) [8,9,10,11]. This fishery is the seventh most important in the world. Furthermore, is considered to be the second most important of the Atlantic Ocean producing more than 6% of the global catch with sardines and mackerel as the commonest captures [12].

In addition to hosting species-specific microsporidia, fish have been reported to carry zoonotic species capable of infecting humans within their gastrointestinal tract. This represents a potential source of cross-contamination in domestic settings, particularly in regions with strong fishing traditions or a high prevalence of recreational fishing. Moreover, wild-caught fish are more likely to harbor zoonotic microsporidia than farmed counterparts [13]. Certain small pelagic species are often consumed whole, and rapid frying may not achieve temperatures sufficient to inactivate the spores. Furthermore, in immuno-compromised individuals, other species may cross the host barrier and cause disseminated infections. For instance, *Trachipleistophora hominis*, a probable insect parasite, has been diagnosed in humans and has even been implicated as a cause of death [14].

Farmed animals are more susceptible to those diseases transmitted by direct cycle, fish being no exception. In the case of microsporidia, most species do not require an intermediate host, hence, with high density of animals, epizooties are prone to occur and easier to notice in aquaculture rather than in wild fish [3]. Therefore, spite the pathology and epidemiology of microsporidia seem to be better understood in farmed fish, epizootic events have been recorded for both [3,15,16]. In addition, these parasites appear to be influenced by temperature, exhibiting seasonal patterns. An increase in the prevalence and severity of lesions caused by microsporidia during summer has been documented in at least two fish species: *Glugea* (*G.*) *stephani* in winter flounder (*Pseudopleuronectes americanus*) and *Loma salmonae* in rainbow trout (*Oncorhynchus mykiss*) [17].

These fungi may cause a variety of symptoms and lesions from very mild to mass mortality. Nevertheless, they can be classified in two major groups: those who cause hypertrophy of single cells (formation of xenomas) and those who do not. Among others, microsporidia can cause in fish: coelom distension, deformities, reduced fertility, muscle liquefaction, impairment swimming, necrosis by pressure miscellaneous organs, reduced growth and larval death [3]. All these symptoms in natural conditions may not be observed since diseased fish are likely predated or brought dead to the laboratory. Therefore, the role of these microorganisms in the natural regulation of population dynamics and associated economic losses in eastern Atlantic is far from being precise.

The aim of this study is to deepen the species of microsporidia in wild caught marketed fish from FAO 34 and their potential effect on economic loss.

## 2. Materials and Methods

### 2.1. Fish Sampling

From 2011 to 2019, various adult fish species were collected from local markets to examine the presence of microsporidia, particularly *Glugea* spp. The specimens were obtained using fishing nets in FAO fishing area 34 (Archipelagos of Madeira and Canary Islands). This region extends from 36°00′ to 26°00′ N latitude and from 20°00′ W longitude to a line drawn from 36°00′ N along 13°00′ W to 29°00′ N, then continuing along a rhumb line in a southwesterly direction to 26°00′ N and 16°00′ W. Fish were caught the same night, immediately placed on ice after capture, and purchased early the following morning.

### 2.2. Dissection

Fish were transported in ice-filled coolers to the Faculty of Veterinary Sciences, University of Las Palmas de Gran Canaria (ULPGC). Prior to dissection, several biometric parameters were recorded, including length, width, and weight. These measurements, along with the post-mortem examination, were performed following standard protocols.

Fulton’s condition factor (K) was used as an index of fish fitness [18]. It was calculated as body mass (g) divided by the cube of body length (cm). During dissection, all major organs were examined for evidence of microsporidian infection. This included gills, brain, heart, gonads, and the lateral muscle, which was sectioned longitudinally and transversely to detect possible xenomas.

Gross lesions were photographed using a Nikon D3300 camera with an 18–55 mm DX lens (Nikon Corporation, Tokyo, Japan), processed in ViewNX-I (Nikon Corporation, Tokyo, Japan), and subsequently edited with Adobe Photoshop 2021 (version 22.5.1, Adobe Inc., San José, CA, USA). Compatible lesions (nodules, masses or cysts) were excised, measured, and squashed to confirm the presence of microsporidian spores. Spore measurements were performed using a Leica DM6000 B microscope (Leica Microsistemas S.L.U., Barcelona, Spain) equipped with Nomarski differential interference contrast (DIC), and a Nikon D3300 attached via a T2 adapter to a photographic tube on a calibrated Nikon YS00. Observations, expressed in micrometers, were made on mature spores obtained from several xenomas. To compare spore morphology, a shape ratio was calculated by dividing the mean spore length by the mean spore width, allowing differentiation between species with more or less elongated spores.

### 2.3. Histology

Thirteen infected round sardinella specimens were selected for histology, including five with early infection (small xenomas) and eight with large xenomas at different stages of maturity. They were fixed in 10% neutral buffered formalin for histopathological examination. Tissue samples were trimmed, dehydrated through a graded ethanol series, passed to xylene, and finally embedded in paraffin wax following standard histological protocols. Serial sections, 5 µm thick, were cut in sets of six using a rotary microtome. Sections 1, 3, and 5 were stained with hematoxylin and eosin (H&E) for general tissue morphology [19], whereas sections 2, 4, and 6 were stained using Kinyoun’s acid-fast method to identify microsporidian spores [20]. Spores that stained positively with Kinyoun’s technique were considered mature. Fish with mild and small lesions were considered in an early stage of infection.

### 2.4. Ultrastructural Study

Xenomas were fixed in 2.5% glutaraldehyde in 0.2 M phosphate buffer (pH 7.2) for more than 8 h. Samples were post-fixed in 1% osmium tetroxide in the same buffer for 4 h, rinsed in fixation buffer, and transferred to 1% aqueous uranyl acetate for 2 h. Subsequently, xenomas were dehydrated through a graded ethanol series (20, 40, 60, 70, 98, and 100%), passed through a 1:1 solution of absolute ethanol and propylene oxide, then pure propylene oxide, followed by a 1:1 mixture of propylene oxide and Embed 812 resin, and finally polymerized in fresh Embed 812 resin for 48 h at 70 °C [21,22].

Semithin sections (1 µm) were obtained using a Leica EM UC7 ultramicrotome and stained with toluidine blue for light microscopy. Ultrathin sections were examined by transmission electron microscopy (TEM) using a Zeiss Sigma 300 VP microscope (Carl Zeiss AG, Oberkochen, Germany). Microscopy analyses were performed at the Advanced Microscopy and Cytometry Service (SIMACe), University of Las Palmas de Gran Canaria, Spain.

### 2.5. Molecular Analysis

Xenomas were stored at −20 °C until DNA extraction. Parasite DNA was extracted using the UltraClean™ Tissue DNA Isolation Kit (Mo Bio Laboratories Inc., Carlsbad, CA, USA) according to the manufacturer’s instructions for animal tissue. The concentration and purity of the extracted DNA were assessed by spectrophotometry, and the DNA was stored at −20 °C until use.

PCR amplification of most of the region encoding the small subunit (SSU) 18S rDNA was carried out in a 25 µL reaction using AmpliTaq^®^ DNA Polymerase (Thermo Fisher Scientific Inc., Waltham, MA, USA) with the primers V1f (5′-CACCAGGTTGATTCTGCC-3′) [23] and HG5F_rev (5′-TCACCCCACTTGTCGTTA-3′) [24]. To amplify the internal transcribed spacer region (ITS) located between the SSU and LSU rDNA, along with adjoining sequences, the primers HG4F (5′-GCGGCTTAATTTGACTCAAC-3′) and HG4R (5′-TCTCCTTGGTCCGTGTTTCAA-3′) were used [25]. PCR conditions followed those described by Casal et al. (2016) [26].

PCR products from xenomas obtained from two different *Sardinella aurita* individuals were purified using a one-step method [27]. Sequencing was performed in both directions with the primers listed above, using the BigDye Terminator v3.1 Cycle Sequencing Kit (Thermo Fisher Scientific Inc.) according to the manufacturer’s instructions. Chromatograms were inspected with FinchTV (v.1.4.0; Geospiza Inc., Seattle, WA, USA). Forward and reverse sequences were aligned to generate consensus sequences using Gene Runner (v.4.0.9.68 Beta; Hastings Software Inc., Hastings, MN, USA). The resulting consensus sequences were compared with those available in GenBank through BLAST (NCBI, Bethesda, MD, USA; https://blast.ncbi.nlm.nih.gov) searches [28] to determine their percent identity to reference sequences.

### 2.6. Phylogenetic Analysis

For the phylogenetic analysis, a tree was constructed using SSU rDNA sequences from 15 microsporidian species retrieved from the GenBank database (accession numbers provided in Table 1), together with the newly generated sequence *Glugea* sp. 53 (MT072043). *Pleistophora typicalis* (AF044387) was used as the outgroup. The sequence of *Glugea cordis* was not included due to the absence of available genetic information. Sequences were aligned as described above, and evolutionary analyses were conducted in MEGA6 [29] using the Maximum Likelihood method based on the Jukes-Cantor model [30]. Positions containing gaps or missing data were excluded from the analysis. Node support was assessed by bootstrap resampling with 1000 replicates.

### 2.7. Statistics

Statistical analyses were conducted using Epi Info™ version 7 (Centers for Disease Control and Prevention, Atlanta, GA, USA). For continuous variables, descriptive statistics included the mean, standard deviation (SD), and range, whereas categorical variables were summarized as frequencies and percentages. Statistical significance was defined as *p* < 0.05.

## 3. Results

### 3.1. Sample Size and Description

During 35. species comprising a total of 576 individuals were analysed and distributed as follows: *Balistes capriscus* (n = 10), *Beryx splendens* (n = 8), *Boops boops* (n = 12), *Coryphaena hippurus* (n = 13), *Chelidonichthys lastoviza* (n = 1), *Dentex dentex* (n = 5), *Dentex gibbosus* (n = 5), *Dentex macrophthalmus* (n = 13), *Diplodus sargus* (n = 10), *Diplodus vulgaris* (n = 6), *Heteropriacanthus cruentatus* (n = 1), *Helicolenus dactylopterus* (n = 3), *Katsuwonus pelamis* (n = 6), *Merluccius merluccius* (n = 1), *Microchirus azevia* (n = 1), *Micromesistius poutassou* (n = 5), *Mullus surmuletus* (n = 11), *Oblada melanura* (n = 2), *Pagellus acarne* (n = 114), *Pagellus erythrinus* (n = 68), *Pagrus auriga* (n = 1), *Pagrus pagrus* (n = 3), *Polyprion americanus* (n = 1), *Pomadasys incisus* (n = 17), *Sardina pilchardus* (n = 25), *Sardinella aurita* (n = 39), *Sarpa salpa* (n = 69), *Scomber colias* (n = 4), *Seriola rivoliana* (n = 1), *Serranus atricauda* (n = 1), *Serranus cabrilla* (n = 21), *Sparisoma cretense* (n = 52), *Sphyraena viridensis* (n = 1), *Spondyliosoma cantharus* (n = 37), *Trisopterus luscus* (n = 5), and *Umbrina canariensis* (n = 4).

### 3.2. Prevalence, Biometrics and Effect on Fish Fitness

Lesions consistent with microsporidian infection were detected in only one fish species, representing 3.3% (19/576) of the examined specimens. All infected individuals were round sardinellas (*S. aurita*), corresponding to a prevalence of 48.7% (19/39). All specimens were collected during the same season and year (2019), September–October. Therefore, the reported prevalence (19/39) corresponds to this single sampling period.

Infected sardinellas had an average weight of 42.11 ± 16.99 g and an average length of 17.90 ± 2.91 cm, whereas non-infected individuals averaged 89.10 ± 35.45 g and 21.58 ± 3.33 cm in length. Thus, parasitized specimens weighed approximately half as much as non-infected ones, and this difference was statistically significant (*p* < 0.001). Differences in length, width, and Fulton’s condition factor between infected and non-infected sardinellas were also statistically significant (Table 2).

### 3.3. Gross Findings

**Figure 1:**
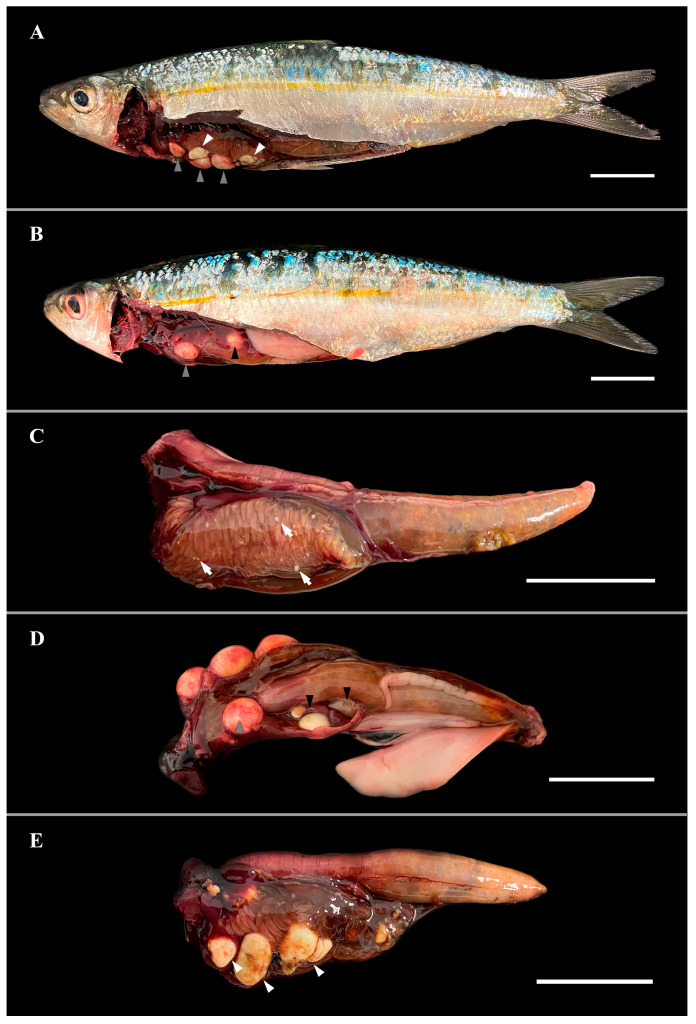
Gross pathology caused by *Glugea* sp., illustrating a probable progression of the disease. (**A**) Sardine with soft, mature, well-vascularized cystic lesions (grey arrowheads) and firm lesions compatible with granulomas undergoing resorption (white arrowheads). (**B**) Transparency of the liver (black arrowhead) reveals a fresh granuloma beneath; grey arrowhead indicates a fresh granuloma not covered by the liver. (**C**) Sardine viscera showing multifocal small white lesions within the mesenchymal tissue of the pyloric caeca (white arrows), this is an example of an early infected fish. (**D**) Viscera of the same sardine shown in panel (**B**); black arrowhead indicates embedded granulomas in the liver parenchyma, consistent with compression-induced necrosis. Note the vasculature surrounding the fresh granuloma (grey arrowhead). (**E**) Sardine viscera with firm lesions compatible with granulomas in resorption (white arrowheads). All scale bars = 2 cm.

The coelomic cavity exhibited mild (1–2 nodules) to severe (<10 nodules) multifocal to coalescing lesions, primarily located on the pyloric caeca but also scattered throughout the cavity. Lesions ranged in size from 0.2 cm to 1.1 cm and affected approximately 5–90% of the cavity (Figure 1). Their morphology and color varied from ovoid to spherical, smooth to wrinkled, white to yellowish, and soft to firm, all consistent with microsporidian infection. Cysts adjacent to the liver produced impressions indicative of pressure-induced necrosis.

### 3.4. Histopathological Findings

**Figure 2:**
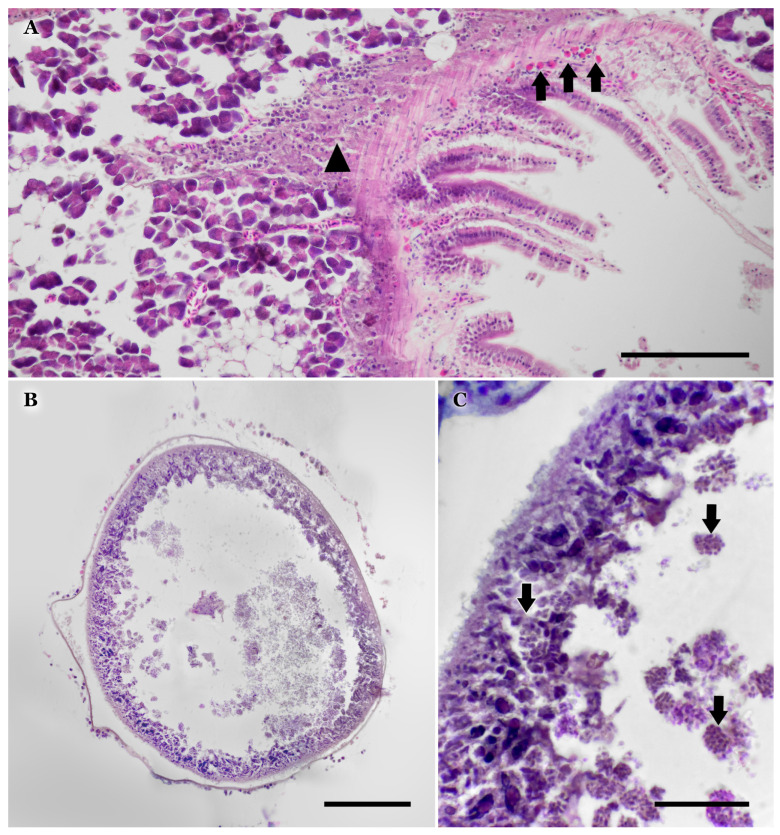
(**A**) Pyloric caecum showing increased amount of eosinophils (black arrows), and associated inflammation and necrosis (black arrowhead). This last lesion is demarcated by capillaries suggesting a possible vasculitis as origin. Scale bar = 100 µm. (**B**) Histology of a xenoma showing mature spores released in the interior along with sporogonies. Scale bar = 100 µm. (**C**) Detail of xenoma wall showing multiple developmental stages, such as sporogonies (black arrows) wich are excreted to the interior of the xenoma. Scale bar = 25 µm.

Histological examination revealed an increased number of eosinophils (up to 10 per field) in certain areas of the pyloric caeca, mainly within the lamina propria and blood vessels of fish in the early stages of infection (Figure 2A). Pancreatic degeneration was also observed in association with these changes (Figure 2A). Pancreatic degeneration was not exclusively linked to these findings; in fish in the early stages of infection, alterations were less severe than those observed in individuals with larger and firmer nodules.

Histological lesions consistent with inmature xenomas were detected in only one fish, likely representing an early stage of infection, with no associated inflammatory response (Figure 2B,C). Within these xenomas, developmental stages were located in the peripheral layer, surrounding a central core of mature spores (Figure 2C).

**Figure 3:**
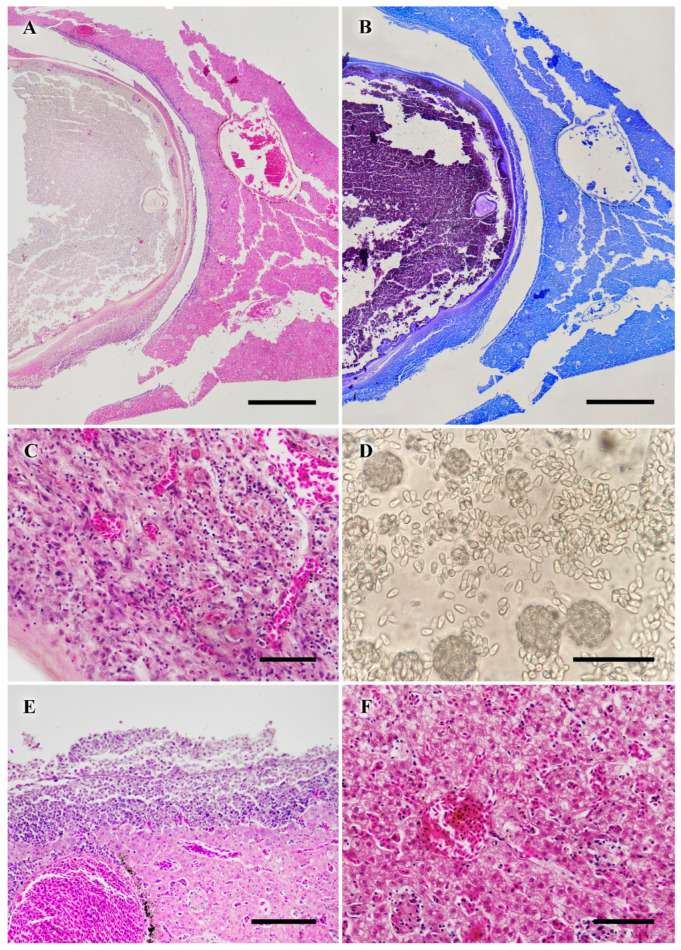
(**A**) Thick-walled “mature xenoma” or granuloma (left) filled with mature spores, which are Kinyoun-positive, and probable reproduction residua. Note the associated inflammation affecting the liver surface (right) as well as the excavation of a concave lesion by pressure necrosis. Scale bar = 600 µm. (**B**) Same as A. Scale bar = 600 µm. (**C**) Detail of the heavily vascularized wall of the “mature xenomas”. Scale bar = 50 µm. (**D**) Released spores in fresh mount. Grouped spores in spheres are regarded as sporogonies. Scale bar = 20 µm. (**E**) Detail of inflamed surface of the liver in touch with the “mature xenoma”. Scale bar = 50 µm. (**F**) Multivacuolar degeneration of the hepatic tissue Scale bar = 50 µm.

Larger cystic lesions consisted of encapsulated mature spores (Kinyoun-positive), which some authors refer to as “mature xenomas” (Figure 3A,B). The cyst wall was composed of mature, well-vascularized granulation tissue (Figure 3C) and, in some cases exhibited active pyogranulomatous inflammation involving heterophils and eosinophils. Phagocytic activity was not observed within the lesions, being restricted to the periphery around mature spores. Spore bundles (sporogonies) were absent in the histology of mature xenomas or granulomas but were observed in fresh mounts (Figure 3D) and in non-inflamed xenomas. Firm, mature lesions did not differ significantly in histological appearance from softer lesions of similar size.

Lesions adjacent to the liver were associated with structural deformation and tissue degeneration, likely related to mechanical pressure from xenomas, as previously reported in fish microsporidiosis. This interpretation is supported by the gross appearance shown in Figure 1 (black arrowheads), where xenomas caused marked deformation and translucency of adjacent hepatic tissue, suggesting structural compromise. In some cases, granulomatous inflammation encapsulating the mass of mature spores extended into hepatic tissue, resulting in adhesion of the cystic granuloma to the organ (Figure 3F).

**Figure 4:**
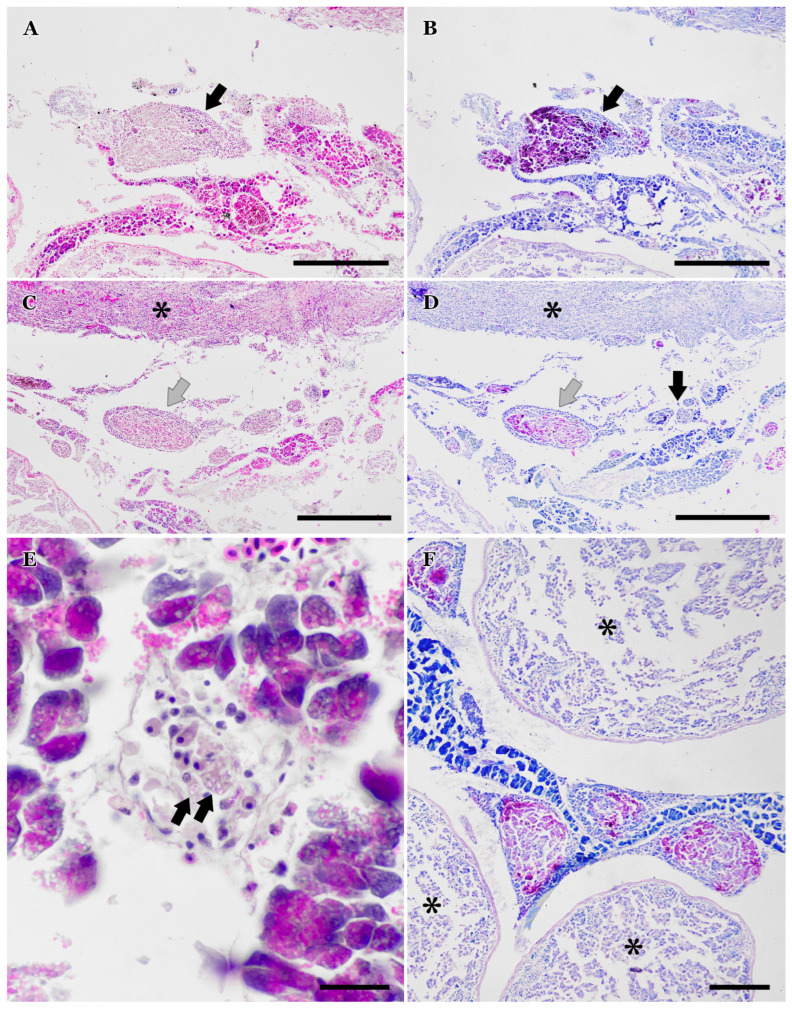
(**A**) Area of recently phagocytosed spores in the coelomic cavity during granuloma formation (arrow) (H&E). Scale bar = 300 µm. (**B**) Same area as panel (**A**), stained with Kinyoun’s method. Note the homogeneous coloration of spores within macrophages. Scale bar = 300 µm. (**C**) Granulomas at different stages: mature (black arrow and asterisk indicating the wall of a ‘mature xenoma’) and chronic-active with layers of leukocytes and fibroblasts (grey arrow). Scale bar = 300 µm. (**D**) Same area as panel (**C**), stained with Kinyoun’s method. Note the peripheral distribution of mature spores in more developed granulomas (black arrow). Scale bar = 300 µm. (**E**) A few macrophages containing spores (arrows), with associated pancreatic alteration and enzyme release. Scale bar = 20 µm. (**F**) Mature granulomas embedded in pancreatic tissue with associated inflammation. Asterisks indicate pyloric caeca. Scale bar = 100 µm.

Multiple granulomas at various stages of maturation were randomly distributed around the pancreas and within connective tissue adjacent to the pyloric caeca (Figure 4A–F). Recent phagocytic activity was clearly identified using Kinyoun stain, with macrophage aggregates appearing Kinyoun-positive (Figure 4A,B). As granulomas developed concentrically, older granulomas exhibited Kinyoun-positive spores only in the outer layers, while central areas were negative (Figure 4C,D,F).

### 3.5. Spores’ Description

**Figure 5:**
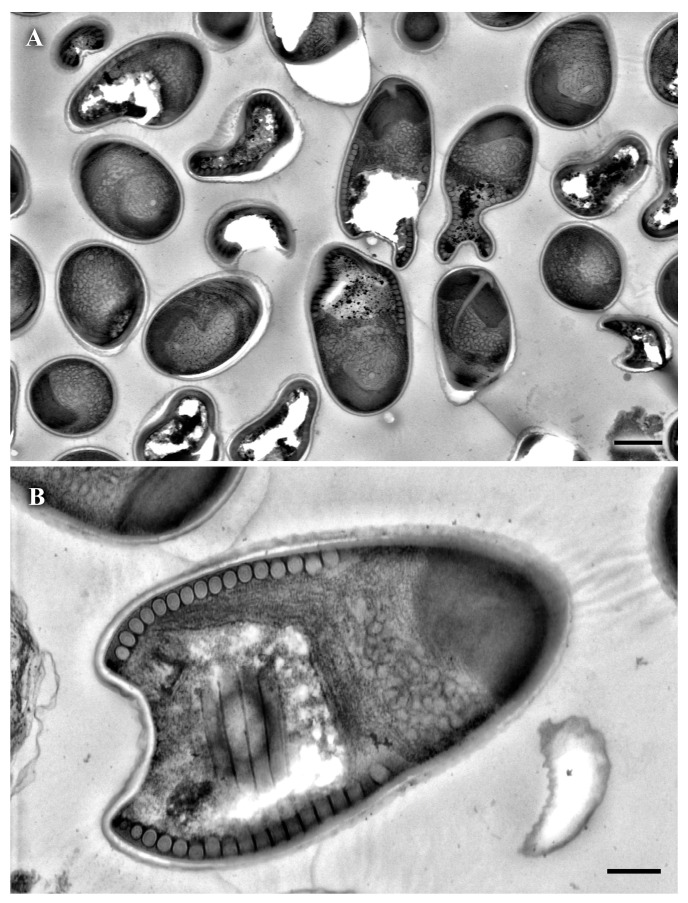
Ultrastructure of *Glugea* sp. from *Sardinella aurita*. (**A**) Scale bar = 1 µm (**B**) Scale bar = 0.4 µm.

In the fresh preparations, the spores appeared as pear-shaped, refractile objects with an apparent vacuole in the thicker end (n = 80). They measured 3.26 ± 0.13 µm in length and 1.79 ± 0.12 µm in width (Table S1). Under transmission electron microscopy (Figure 5), spores appeared more ellipsoidal than pear-shaped, with few aberrant forms, probably artefacts. The capsule thickness measured 66.4 ± 20.7 (n = 11), ranging from a minimum of 36 nm to looser regions up to 98 nm. The diameter of the anchoring disc was 576.5 ± 44.8 (n = 6), with a thickness of 139.8 ± 14.6 nm. The posterior vacuole occupied nearly half of the spore, while the remaining space contained the anterior and posterior polaroplast. The polar filament formed 14 to 15 coils.

### 3.6. Molecular Analysis

Analysis of nearly complete SSU rDNA, ITS, and partial LSU rDNA sequences obtained from spores isolated from xenomas revealed no differences between the two sequences generated in this study. A 1679 bp sequence was deposited in GenBank under accession number MT072043.

A BLAST search of the SSU rDNA fragment (1264 bp) showed strong similarity to microsporidia infecting fish, all belonging to the genus *Glugea*. The highest identity percentages with 100% query coverage were: 100% identity to *Glugea* sp. GReina-2025a (KY882286) and *Glugea* sp. ST1 (OR733697), both previously misclassified as *G. plecoglossi*. Additional close matches included *G. thunni* (OM914139; 99.92%), *G. hertwigi* (GQ203287; 99.53%), *G. plecoglossi* (AB623035; 99.21%), and *Glugea* sp. CCG1 (KU885382; 99.13%). Other *Glugea* sequences showed >99% identity but with lower query coverage (73–98%), such as *G. stephani*, *G. gasterostei*, *G. atherinae*, and additional *Glugea* sp. clones (Table S2).

Due to differences in query coverage, several *Glugea* species exhibited lower sequence similarity to the sample analyzed in this study compared to another microsporidian genus. For instance, *G. jazanensis* (KP262018) showed 86.3% identity, *G. sardinellensis* (KU577431) 78.7%, and *G. pagri* (JX852026) 77.6%, whereas *Pleistophora typicalis* (AF044387) displayed 87.8% identity. Consequently, the 16S rDNA sequences were trimmed to 700 bp and reanalyzed. This adjustment provided clearer separation between the outgroup and additional *Glugea* species, which exhibited even lower similarities in the initial comparison. Notably, the identity for *G. sardinellensis* (KU577431) increased from 78.7% to 96.2%, and for *G. pagri* (JX852026) from 77.6% to 99.5%, while the similarity to *Pleistophora typicalis* (AF044387) decreased from 87.8% to 85.5%. Considering this adjustment, the species ranking above 99% similarity, in decreasing order, were as follows: *Glugea* sp. GReina-2025a (KY882286), 100%; *Glugea* sp. ST1 (OR733697), 100%; *G. thunni* (OM914139), 100%; *G. hertwigi* (GQ203287), 100%; *Glugea* sp. CSl-2020a clone 8 (MT680622), 99.8%; *Glugea* sp. CSl-2020a clone 9 (MT680621), 99.8%; *Glugea* sp. ST2 (OR722585), 99.8%; *G. gasterostei* (KM977990), 99.8%; *Glugea* sp. CBG1 (KU885381), 99.6%; *G. atherinae* (U15987), 99.6%; *G. stephani* (AF056015), 99.6%; *G. pagri* (JX852026), 99.5%; *G. anomala* (AF044391), 99.2%; and *G. plecoglossi* (AB623035), 99.0%. A similar analysis was done but in this case analysing 1061 bp giving similar results as before. However percentage similarities decreased from 100% to 99.9% for *G. thunni* (OM914139) and 99.5 for *G. hertwigi* (GQ203287) (Table 3 and Table S3). In Table 3 and Table S4 a 1061 nucletides sequences were analized resulting in similar conclussions.

For greater clarity, the internal transcribed spacer region and partial 23S rDNA of our sequence were compared with homologous sequences available in GenBank. The results showed 100% identity between *Glugea* sp. GReina-2025a (KY882286) and *G. thunni* (OM914139); 98.3% for *Glugea* sp. CBG1 (KU885381); 98.0% for *G. plecoglossi* sequence (AJ295326); 97.6% for *G. hertwigi* (GQ203287); and 96.4% for *G. anomala* (AF044391) (Table 3 and Table S5).

Finally, the overall similarity based on the alignment of partial 16S rDNA, the internal transcribed spacer region, and partial 23S rDNA sequences (1686 bp) confirmed 100% identity with *Glugea* sp. GReina-2025a (KY882286) and 99.9% identity with *G. thunni* (OM914139). The remaining sequences fell below 98.7%, including *Glugea* sp. CCG1 (KU885382.1) at 98.6%, *G. hertwigi* (GQ203287) at 98.8%, and *G. anomala* (AF044391) at 98.1%. (Table 3 and Table S6).

### 3.7. Phylogenetic Analysis

**Figure 6:**
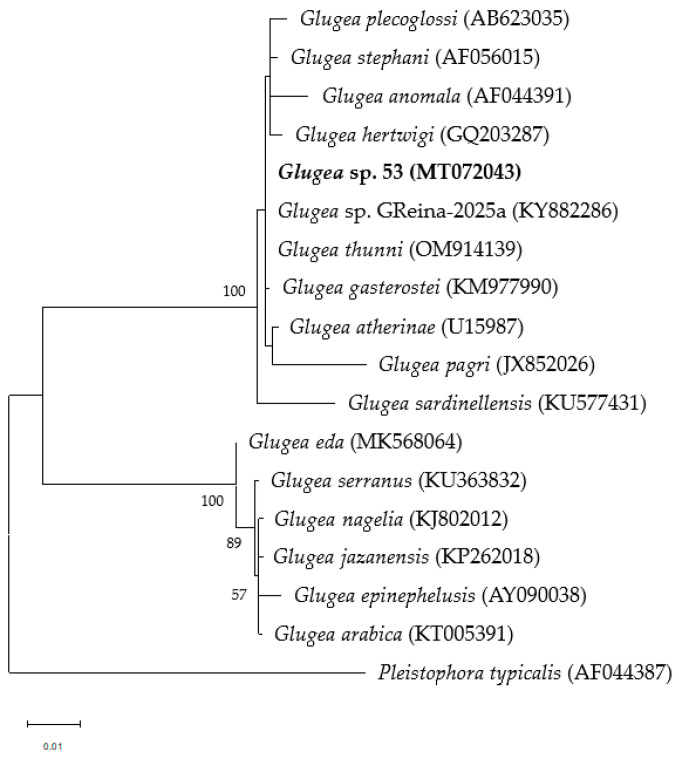
Phylogenetic relationships of the *Glugea* sp. 53 found in *S. aurita* spp. inferred from the small subunit (SSU) rDNA dataset of selected microsporidian sequences using the Maximum Likelihood method and Jukes-Cantor model [27]. Gen Bank accession numbers are reported in parentheses. There were a total of 1252 positions in the final dataset. Bootstrap probabilities (1000 replicates) are given in branches (%). *Pleistophora typicalis* was used as the outgroup. Scale bar shows the number of changes per site.

Phylogenetic analysis of SSU rDNA using maximum likelihood indicated that the novel sequence grouped within the first *Glugea* clade (Figure 6), displaying <4% variability compared with other members, whereas a second *Glugea* clade clustered separately with 100% bootstrap support and showed >9% divergence from the sequence obtained in this study (Figure 6).

## 4. Discussion

Despite numerous reports highlighting the significance of microsporidia in commercially important fish species, a low infection rate was observed in this study, with only 1 out of 35 fish species found to be infected. Among the fish species examined, previous studies have reported microsporidian infections, typically with low prevalence rates (approximately 10–20%). These include various *Glugea* species such as *G. machari* in *Dentex dentex* (Rab Island, Croatia), *G. cordis* in *Sardina pilchardus* (Mediterranean Sea, France), *G. shiplei* in *Trisopterus luscus* (English Channel, England) [31], *G. serranus* in *Serranus atricauda* (Madeira, Portugal) [11], and *G. sardinellensis* in *S. aurita* (Mediterranean Sea, Tunisia) [32]. Other microsporidian species reported in fish sampled in this study include *Pleistophora finisterrensis* from *Micromesistius poutassou* (northwest Spain) [33], *Loma boopsi* from *Boops boops* (Senegal) [9], *Loma diplodae* from *Diplodus sargus* (Mediterranean Sea, France) [34] as well as several undescribed microsporidia from *Pagrus pagrus*, *Umbrina canariensis* (Senegal) [8] or *Scomber colias* (Morocco) [10]. Although infection was detected only in round sardinella, the absence of microsporidia in 34 other species examined provides critical epidemiological insight. These negative results challenge the perception that xenoma-forming microsporidia are widespread threats in FAO Area 34 and underscore the importance of reporting absence data in prevalence studies.

Interestingly, the only fish species found to be positive to microsporidia in this study, the round sardinellas (*S. aurita*), exhibited a prevalence comparable to that observed in aquaculture fish (approximately 50%). For example, *Glugea pagri* showed a prevalence of 54% in farmed *Pagrus major* in China [35]. Therefore, the *Glugea* species identified in this study appears to be a significant pathogen for round sardinellas in their natural environment. In fact, during a period of elevated water temperatures in the Mediterranean Sea, a mass mortality event of round sardinellas occurred, with a *Glugea* species implicated as the causative agent [15,16]. This species is genetically very similar to the *Glugea* described in the present study, and it is likely that the combination of high temperatures and a weakened immune response allowed the parasite to proliferate and ultimately kill its hosts.

Numerous studies have reported that microsporidiosis can severely affect host fitness [36,37,38]. For instance, Atlantic salmon infected with *Desmozoon lepeophtherii* presented a 20–25% reduction in body weight [39], and *G. sardinellensis* infections have been more frequently recorded in smaller fish [32]. In the present study, infected individuals of *S. aurita* weighed nearly half as much as non-infected counterparts. These parasitized fish were also shorter in length and width, resulting in lower Fulton’s condition index values, confirming that this *Glugea* species can negatively affect the growth of *S. aurita* and potentially contribute to juvenile mortality. Although age determination was not performed, all specimens were collected during the same fishing season from local markets, where fish are generally sorted by size for commercial purposes. This reduces the likelihood of major age differences among the sampled individuals.

In commercial fisheries, infected fish are often sold already gutted, which prevents rejection by consumers and allows them to be marketed regardless of infection status. However, the substantial reduction in fish weight translates into a lower market value—typically about half the usual price per unit—and overall reduced profitability.

The pathological changes observed in this study may provide insight into the infection pathway of *S. aurita* by this *Glugea* species. Necrotic lesions associated with eosinophil infiltration in the pyloric caeca may represent potential entry points for the parasite, as suggested for other microsporidian infections. In other fish species, prior to organ colonization, intestinal xenomas have been observed within the lamina propria and muscularis layers, where they may access the bloodstream [40,41]. Infections by other microsporidian species often involve systemic transport and vasculitis [41,42], which may also apply to *Glugea* species. Although no definitive evidence of vasculitis was found in the histological sections of infected fish, its presence cannot be ruled out.

The specific target cells of this microsporidian remain unidentified, although the parasite is believed to infect cells in the connective tissues within the coelomic cavity. Consistent with previous studies, the early stages of infection appear to occur without associated inflammation [40], further supporting the hypothesis of a stealthy initial invasion followed by systemic dissemination. Additionally, eosinophil identification was based on morphology and location, primarily within blood vessels, and healthy tissue was used for comparison. We acknowledge that eosinophilic granular cells (EGCs) are common in some teleosts and resemble eosinophils; however, EGCs are typically located in the intestinal submucosa, whereas the observed cells were intravascular. This distinction supports their identification as eosinophils in this study [43].

During xenoma development, numerous spores accumulate within the host cell, which eventually ruptures, releasing spores and triggering an immune response. Leaked spores in the coelomic cavity may be phagocytosed and digested by macrophages, forming granulomas characterized by a central core of more digested spores (Kinyoun-negative) surrounded by outer layers of recently phagocytosed spores (Kinyoun-positive). As the infection progresses, granulomatous inflammation encapsulates the xenomas, forming cystic granulomas primarily composed of unphagocytosed mature spores [40].

The transmission route to other hosts remains unclear. Two hypotheses may explain its spread: (1) horizontal transmission via ingestion of infected fish—dead or alive—similar to some terrestrial coccidia [44], or (2) vertical transmission through infected eggs, as reported in other microsporidian species such as *Ovipleistophora* in golden shiners (*Notemigonus crysoleucas*), *Glugea* spp. in mottled sculpins (*Cottus bairdii*) and ayu fish (*Plecoglossus altivelis*), and *Encephalitozoon* in rabbits [40,42,45,46]. Although no gross lesions compatible with xenomas were found in the gonads in this study, this remains an important consideration for future studies. To better understand the parasite’s entry route and pathogenesis, further histochemical staining and experimental infections are needed.

Microsporidian taxonomy is typically based on host specificity, infection site, geographic locality, and morphological/molecular data. However, due to incomplete datasets, species identification can be challenging, with morphologically similar species found in different hosts and distant locations. Within the family Clupeidae, two *Glugea* species have been described: *G. sardinellensis* in *S. aurita* [33] and *G. cordis* in *Sardina pilchardus* [47]. The specimens analyzed in this study match the spore measurements reported for *G. cordis* but differ in host species, lack ultrastructural and genetic data, and the original description of *G. cordis* notes tear-shaped spores. Conversely, *S. aurita* is the type host for *G. sardinellensis*, yet the material in this study differs genetically and morphologically, underscoring the importance of genetic and ultrastructural analyses for accurate species identification. Based on available data, the species described here is most consistent with *G. thunni* [48]. Originally described from a single farm-raised bluefin tuna (*Thunnus thynnus*) in the Mediterranean Sea, *G. thunni* caused a massive infection likely responsible for host mortality. The tuna was reportedly fed sardines and sardinellas, suggesting that *S. aurita* may be the true type host and that the infection in tuna was opportunistic. Additionally, five unnamed records of *Glugea* in *S. aurita* exist (OR733697, MT68062, OR722585, MT680622 and PP864450) and one from *Sardina pilchardus* (KY882286), three of which were misidentified as *G. plecoglossi* (OR733697, OR733697 and OR722585). All these mentioned glugeas are placed withing the Group 2 sensu Mansour et al. (2016) [33] close related to *G. thunni*. The differences in sequence varied from 0 to 3 comparing a partial sequence of the 16S rDNA. However, the divergence between other glugeas is also low and it has been suggested to sequence new molecular markers to clarify the phylogenetic of this group [16].

Morphologically, spores from bluefin tuna measured 3.1–4.5 × 1.8–2.5 µm (shape ratio ≈ 1.80), while those from *S. aurita* (*Glugea* sp. 53) measured 2.9–3.6 × 1.6–2.1 µm (shape ratio ≈ 1.76). Therefore, the spores described in this paper are slightly shorter but similarly shaped. The tuna also exhibited a second, more elongated spore type (shape ratio ≈ 3), which was not observed in this study. Polar filament coils were arranged in a single row, with 13–14 coils in tuna and 14–15 in round sardinella, although one spore in the original *G. thunni* description showed 15 coils [48]. These differences in spore size may reflect host-specific developmental variation.

Two previously reported *Glugea* records from *S. aurita* in the Mediterranean Sea are genetically close to *G. thunni* but were not identified as such. These records describe smaller xenomas in smaller fish and spores that are larger and slightly less elongated (3.9–4.6 × 2.3–2.8 µm, shape ratio ≈ 1.67 [16]; 3–5 µm in length [15]) compared to the spores observed in this study (2.9–3.6 × 1.6–2.1 µm, shape ratio ≈ 1.76). These records are more similar to each other than to the *G. thunni* spores from bluefin tuna, which are more elongated and include a second spore type. This further supports the hypothesis that *S. aurita* is the primary host for *G. thunni* and underscores the need for comprehensive morphological and genetic characterization to resolve microsporidian taxonomy.

Considering the observed genetic and morphological differences between hosts and *Glugea* species, the sequences from round sardinella and bluefin tuna may represent a complex of cryptic *Glugea* species, warranting further molecular investigation. However, an alternative hypothesis is that all Mediterranean and Northeastern Atlantic records, including the microsporidian analyzed in this study, correspond to a single species—*G. thunni*—which would indicate its role as a significant pathogen for *S. aurita* with occasional infections in other hosts. A third possibility is ongoing speciation, with populations diverging into distinct taxa associated with different geographic regions. The similarity between *G. thunni* and species such as *G. hertwigi*, despite their distant hosts, further suggests that these lineages may represent geographically structured variants within *Glugea*, highlighting the need for additional molecular markers to clarify whether these taxa form a single species or a broader species complex.

Although morphology alone is insufficient for definitive species identification, the strong resemblance between *G. cordis* and the specimens described in the two Mediterranean records is notable, particularly in terms of shape ratio and spore dimensions. However, *G. cordis* is originally described with tear-shaped spores, differing from the rounded spores observed in this study. This discrepancy highlights the need for renewed sampling of microsporidia from its type host, *Sardina pilchardus*, to potentially redescribe *G. cordis*. Such data could clarify its taxonomic relationship within the genus *Glugea* and help determine whether it represents the correct name for this cryptic species complex, or whether it is a distinct microsporidian altogether.

## 5. Conclusions

This study identifies a microsporidian species infecting wild *S. aurita* for the first time in the Eastern Central Atlantic, genetically consistent with *G. thunni*, and reveals its significant impact on host fitness and fishery economics. Infected fish showed reduced weight, length, and condition factor, leading to considerable economic losses. The parasite’s genetic similarity to other *Glugea* records supports the hypothesis that *S. aurita* may be the true host of *G. thunni*, though cryptic speciation cannot be ruled out. Further research is needed to clarify transmission routes, host specificity, and taxonomy.

## Figures and Tables

**Table 1:** Microsporidian species used for multiple sequence alignment and phylogenetic tree construction, along with their GenBank accession numbers, host species, habitat and source.

Microsporidian Species	Accession Numbers	Host	Habitat	Source
*Glugea* sp. 53	MT072043	*Sardinella aurita*	Marine	Present study
*Glugea* sp. GReina-2005a	KY882286	*Sardina pilchardus*	Marine	GenBank
*Glugea* sp. ST1	OR733697	*Sardinella aurita*	Marine	GenBank
*Glugea* sp. ST2	OR722585	*Sardinella aurita*	Marine	GenBank
*G. anómala*	AF044391	*Gasterosteus aculeatus*	Marine	GenBank
*G. arabica*	KT005391	*Epinephelus polyphekadion*	Marine	GenBank
*G. atherinae*	U15987	*Atherina boyeri*	Marine	GenBank
*G. eda*	MK568064	*Caesio striata*	Marine	GenBank
*G. epinephelusi*	AY090038	*Epinephelus akaara*	Marine	GenBank
*G. gasterostei*	KM977990	*Gasterosteus aculeatus*	Marine	GenBank
*G. hertwigi*	GQ203287	*Osmerus mordax*	Marine	GenBank
*G. jazanensis*	KP262018	*Lutjanus bohar*	Marine	GenBank
*G. nagelia*	KJ802012	*Cephalopholis hemistiktos*	Marine	GenBank
*G. pagri*	JX852026	*Pagrus major*	Marine	GenBank
*G. plecoglossi*	AB623035	*Plecoglossus altivelis*	Freshwater	GenBank
*G. sardinellensis*	KU577431	*Sardinella aurita*	Marine	GenBank
*G. serranus*	KU363832	*Serranus atricauda*	Marine	GenBank
*G. stephani*	AF056015	*Pleuronectes americanus*	Marine	GenBank
*G. thunni*	OM914139	*Thunnus thynnus*	Marine	GenBank
*Pleistophora typicalis*	AF044387	*Myoxocephalus scorpius*	Marine	GenBank

**Table 2:** Round sardinella morphometric measurements. SD: Standard deviation.

	Mean	SD	Minimum	Median	Maximum	*p* Value
Weight Total (g)	66.21	36.49	29.88	51.36	142.91	
Parasitized	42.11	16.99	29.88	35.20	103.77	
Non-parasitized	89.10	35.45	30.12	91.61	142.91	<0.001 *
Total Length (cm)	19.79	3.61	15.00	18.50	29.00	
Parasitized	17.90	2.91	15.50	17.50	29.00	
Non-parasitized	21.58	3.33	15.00	21.50	26.50	<0.001 *
Width (cm)	8.60	1.83	5.50	8.50	11.50	
Parasitized	7.59	1.37	5.50	8.00	9.50	
Non-parasitized	9.55	1.72	6.00	10.06	11.50	<0.001 *
Fulton index (g/cm^3^)	0.7873	0.1264	0.4255	0.7733	1.1822	
Parasitized	0.7278	0.1194	0.4255	0.7480	0.9208	
Non-parasitized	0.8439	0.1075	0.7354	0.8196	1.1822	<0.004 *

* Statistically significant.

**Table 3:** Similarity percentages of partial sequences of the 16S and 23S rDNAs, as well as a 1686 bp sequence comprising nearly the complete 16S rDNA and the full internal transcribed spacer (ITS), between Glugea sp. 53 (MT072043) and other Glugea species.

Glugea	16S rDNA (700 bp) (1061 bp)		ITS-23S rDNA (417 bp)	16S-ITS-23S (1686 bp)
*Glugea* sp.* GReina-2025a (KY882286)	100	100	100	100
*Glugea* sp.* ST1 (OR733697)	100	100	-	-
*G. thunni* (OM914139)	100	99.9	100	99.9
*G. hertwigi* (GQ203287)	100	99.5	97.6	98.8
*G. gasterostei* (KM977990)	99.8	99.8	-	-
*Glugea* sp. CSl-2020a clone 9 (MT680621)	99.8	99.8	-	-
*Glugea* sp.* ST2 (OR722585)	99.8	99.8	-	-
*Glugea* sp. CSl-2020a clone 8 (MT680622)	99.8	99.7	-	-
*G. stephani* (AF056015)	99.6	99.7	-	-
*G. atherinae* (U15987)	99.6	99.7	-	-
*G. pagri* (JX852026)	99.5	-	-	-
*G. anomala* (AF044391)	99.2	99.0	96.4	98.1
*G. sardinellensis* (KU577431)	96.2	-	-	-
*G. plecoglossi* (AB623035)	99.0	99.1	-	-

* Previously misclassified as *G. plecoglossi.*

## Data Availability

The original contributions presented in this study are included in the article/Supplementary Material. Further inquiries can be directed to the corresponding author.

## Supplementary Materials

The following supporting information can be downloaded at: https://www.mdpi.com/article/10.3390/vetsci12121121/s1, references [49,50,51,52,53,54,55,56,57,58,59,60,61,62,63,64,65,66,67,68,69,70] are cited in the Supplementary Materials file. Table S1: Epidemiology, size of xenoma and spores of *Glugea* sp. spores compared to other species from the same genus; Table S2: Similarities and differences among representatives of the genera *Glugea* and *Pleistophora* for 16S rDNA sequences. Percent sequence identity percentage (below the diagonal) and pairwise nucleotide differences (above the diagonal) is based on alignment of 1252 positions. Results between *Glugea* sp. 53 and the other microsporidia are shown in bold.; Table S3: Similarities and differences among representatives of the genera *Glugea* and *Pleistophora* for 16S rDNA sequences. Percent sequence identity percentage (below the diagonal) and pairwise nucleotide differences (above the diagonal) is based on alignment of 700 positions. Results between *Glugea* sp. 53 and the other microsporidia are shown in bold.; Table S4: Similarities and differences among representatives of the genus Glugea for 16S rDNA sequences. Percent sequence identity percentage (below the diagonal) and pairwise nucleotide differences (above the diagonal) is based on alignment of 1061 positions. Results between Glugea sp. 53 and the other microsporidia are shown in bold; Table S5: Similarities and differences among representatives of the genera *Glugea* and *Pleistophora* for Internal transcribe region and partial 23S rDNA sequences. Percent sequence identity percentage (below the diagonal) and pairwise nucleotide differences (above the diagonal) is based on alignment of 417 positions. Results between *Glugea* sp. 53 and the other microsporidia are shown in bold.; Table S6: Similarities and differences among representatives of the genera *Glugea* and *Pleistophora* for partial 16S rDNA, Internal transcribe region and partial 23S rDNA sequences. Percent sequence identity percentage (below the diagonal) and pairwise nucleotide differences (above the diagonal) is based on alignment of 1686 positions. Results between *Glugea* sp. 53 and the other microsporidia are shown in bold.

## Abbreviations

The following abbreviations are used in this manuscript:

µm	Micrometer
mm	milimeter
bp	Base pairs
