# Supplementary material for: On the Epidemiology of Xenoma-Forming Microsporidia in Wild Caught Fish from Macaronesia (FAO34)
Source: Vet Sci. 2025 Nov 26;12(12):1121. doi: 10.3390/vetsci12121121 (PMC12737635; doi:10.3390/vetsci12121121)
Supplement: Supplementary file 1 [file vetsci-12-01121-s001.zip › vetsci-3991314-supplementary.pdf]

veterinary sciences

MDPI

Article

On the epidemiology of xenoma-forming microsporidia in wild caught fish from Macaronesia (FAO34)

Kevin M. Santana-Hernández [1], Eva Betancor [2], Ana Sofía Ramírez [1]*, Begoña Acosta [1], Miriam Rodríguez [1], Emilio Soler-Onís [3], José Pestano [2] and Eligia Rodríguez-Ponce [1]

[1] Department of Pathology, Faculty of Veterinary Science, Universidad de Las Palmas de Gran Canaria (ULPGC), Las Palmas, Spain; kevin.santana106@alu.ulpgc.es, anasofia.ramirez@ulpgc.es, bego.acosta@ulpgc.es, eligia.rodriguezponce@ulpgc.es
[2] Genetic Laboratory, ULPGC, Las Palmas de Gran Canaria, Spain; eva.betancor@ulpgc.es, jose.pestano@ulpgc.es
[3] Banco Español de Algas, FPCT de la Universidad de Las Palmas, Spain; esoler@marinebiotechnology.org
* Correspondence: anasofia.ramirez@ulpgc.es; Tel.: +34 928457432

 https://doi.org/10.3390/vetsci12121121

Table S1. Epidemiology, size of xenoma and spores of *Glugea* sp. spores compared to other species from the same genus.

Glugea sp.	Localization in host	Habitat	Geographical distribution	Host	Xenoma (mm)	Spores (µm)	Shape index	Accession numbers	References
Glugea sp. 53	Connective tissue in coelomic cavity	Marine	Atlantic Ocean, Canary Islands, Spain	*Sardinella aurita*	2-11	2.9-3.6 × 1.6-2.1	1.76	MT072043	**This study**
G. stephani	Connective tissue	Marine	Atlantic Ocean	*Pleuronectes americanus*	0.25-2	5 × 2.8	1.79	AF056015	[49]
G. berglax	Wall of mid-intestine and gall-bladder	Marine	Atlantic Ocean, Newfoundland, Canada	*Macrourus berglax*	0.5	4.2-8 × 2.4-3.1	2.17	-	[50]
G. hertwigi	Submucosa gastrointestinal and mesentery	Marine	Atlantic Ocean, Prince Edward Island, Canada	*Osmerus mordax*	0.2-5	3.5-5.5 × 1.5-2.6	2.22	GQ203287	[51]
G. heraldi	Subcutaneous tissue	Marine	Atlantic Ocean, Florida, USA	*Hippocampus erectus*	0.1-0.8	3.6-4.5 × 1.8-2.3	1.98	-	[52]
G. acuta	Connective tissue of the dorsal fin muscle	Marine	Atlantic coast of France	*Synganthus acus, Nerophis (Entelurus) aequorus*	-	5 × 3-3.5	1.67	-	[47]
G. shiplei	Skeletal muscle and gastrointestinal lamina muscularis	Marine	Atlantic Ocean, English channel	*Trisopterus luscus*	5 × 3	3.5 × 2.5	1.40	-	[53]
G. gasterostei	Abdominal cavity	Marine	Baltic Sea, Gulf of Finland	*Gasterosteus aculeatus*	4	4.8-6.0 × 2.1-2.8	2.21	KM977990	[54]
G. destruens	Skeletal muscles	Marine	Atlantic Ocean, France and South Africa	*Callionymus lyra*	-	3-3.5 × 2-2.5	1.45	-	[47], [55]
G. capverdiensis	Intestinal wall, mesentery and ovary	Marine	Atlantic Ocean, Cape Verde	*Myctophum punctatum*	Up to 2	3.6-4.8 × 1.8-2.6	1.92	-	[56]
G. serranus	Connective tissue	Marine	Atlantic Ocean, Madeira	*Serranus atricauda*	10	6-7 × 2.8-4	1.95	KU363832	[11]
G. anómala	Visceral cavity.	Marine	Mediterranean Sea	*Gasterosteus aculeatus*	4	4.5 × 2.3	1.96	AF044391	[57]
G. atherinae	Body cavity	Marine	Mediterranean Sea	*Atherina boyeri*	-	4.5-6.5 × 2.6-3.3	1.85	U15987	[58]
G. cordis	Connective tissue and heart muscular fibres	Marine	Mediterranean Sea	*Sardina pilchardus*	-	3-3.5 × 2	1.50	-	[48]
G. depressa	Liver	Marine	Mediterranean Sea	*Coris julis*	-	4.5-5 × 1.5-2	2.75	-	[47]
G. machari	Liver	Marine	Mediterranean Sea, Croatia	*Dentex dentex*	0.3-0.4 × 0.25-0.28	3-4.5 × 0.8-1.5	3.38	-	[59]
G. plecoglossi s.l.	-	Marine	Spain	*Sardina pilchardus*	-	-	-	KY882286	Unpublished
G. sardinellensis	Connective tissue	Marine	Mediterranean Sea	*Sardinella aurita*	1-16	5-5.5 × 2.5-3	1.92	KU577431	[32]
G. thunni	Body cavity	Marine	Mediterranean Sea	*Thunnus thynnus*	0.2-7.5	≈ 3,1 - 4,5 × 1,8-2,5; 5,9-6,8 × 1,6 - 2,7	1.8 or 3	OM914139	[48]

G. arabica	Intestinal epithelium	Marine	Red Sea, Saudi Arabia	*Epinephelus polyphekadion*	up to 1.0	5.9-6.6 × 2.9-3.7	1.91	KT005391	[60]
G. eda	Body cavity	Marine	Red Sea, Saudi Arabia	*Caesio striata*	3-5	4-6 × 2-3	2.00	MK568064	[61]
G. jazanensis	Skeletal muscles of body cavity and mesenteric tissues	Marine	Red Sea, Saudi Arabia	*Lutjanus bohar*	2-5	4.0-4.8 × 2.0-2.5	1.96	KP262018	[62]
G. nagelia	Intestinal wall	Marine	Red Sea	*Cephalopholis hemistiktos*	-	4.3-6.0 × 1.8-2.9	2.23	KJ802012	[63]
G. bychowskyi	Intestinal wall and testes	Freshwater and Marine	Caspian Sea	*Alosa kessleri volgensis*	-	3.6 × 1.8	2.00	-	[59]
G. shulmani	Intestinal wall	Marine	Caspian sea	*Neogobius caspius, N. fluviatilis pallas and N. melanostomus affinis*	0.018-0.080	2.2-2.4 × 1.2-1.6	1.67	-	[59]
G. luciopercae	Submucosa of intestine, mesenteries, ovary and gills.	Freshwater and Marine	Caspian and Aral Seas	*Stizostedion lucioperca, Clupeonella delicatula caspia and C. cultiventris cultiventris*	-	3.8-4.8 × 1.7-2.4	2.10	-	[64]
G. nemipteri	Liver, gonads and smooth muscles.	Marine	Bay of Bengal, Indian Ocean	*Nemipterus japonicus*	8-12	5.5-6 × 4.5-5	1.21	-	[65]
G. epinephelusi	Body cavity	Marine	South China Sea	*Epinephelus akaara*	-	4.6-7.2 × 2.8-3.5	1.85	AY090038	[66]
G. pagri	Intestinal muscularis externa or submucosa	Marine	Gulf of Daya, South China Sea	*Pagrus major*	up to 2	3.9-5.1 × 2.1-3.0	1.78	JX852026	[35]
G. vicentiae	Subcutaneous tissue	Marine	Pacific Ocean, Southern Australia	*Vincentia conspersa*	1-2	5.1 × 2.2	2.32	-	[67]
G. punctifera	Connective tissue of ocular muscles	Marine	Atlantic coast of France, Okhotsk Sea and Sea of Japan	*Pollachius virens, Theragra chalcogramma*	-	4-5 × 3	1.33	-	[47], [68]
G. plecoglossi	Connective tissue	Freshwater	China	*Plecoglossus altivelis*	2	4.8-6.2 × 2.4-3	2.03	KX814862	[69]
G. plecoglossi	Viscera	Freshwater	Japan	*Plecoglossus altivelis*	-	-	-	AJ295326	[70]
G. plecoglossi	-	Freshwater	Japan	*Plecoglossus altivelis*	-	-	-	AB623035	Unpublished
Glugea sp. CBG1	Viscera and body wall	Freshwater	New Hampshire, USA	*Cottus cognatus*	0.5-10	5.24 × 2.35	2.23	KU885381	[46]
Glugea sp. CCG1	Viscera and body wall	Freshwater	New Hampshire, USA	*Cottus bairdii*	0.5-10	5.24 × 2.35	2.23	KU885382	[46]

Table S2. Similarities and differences among representatives of the genera *Glugea* and *Pleistophora* for 16S rDNA sequences. Percent sequence identity percentage (below the diagonal) and pairwise nucleotide differences (above the diagonal) is based on alignment of 1252 positions. Results between *Glugea* sp. 53 and the other microsporidia are shown in **bold**.

	Species (Accession number)	1	2	3	4	5	6	7	8	9	10	11	12	13	14	15	16	17	18
1	***Glugea* sp. 53 (MT072043)**		**0**	**1**	**6**	**10**	**18**	**28**	**51**	**57**	**94**	**97**	**99**	**100**	**128**	**167**	***259***	***273***	**150**
2	*Glugea* sp GReina-2025a (KY882286)	**100**		1	6	10	18	28	51	57	94	97	99	100	128	167	259	273	150
3	*Glugea thunni* (OM914139)	**99.9**	99.9		6	10	18	27	50	57	93	96	98	99	127	166	258	273	149
4	*Glugea hertwigi* (GQ203287)	**99.5**	99.5	99.5		14	22	33	56	60	96	99	101	102	130	169	264	276	155
5	*Glugea plecoglossi* (AJ295326)	**99.1**	99.1	99.1	98.8		26	37	60	64	99	102	104	105	133	172	268	281	156
6	*Glugea anomala* (AF044391)	**98.5**	98.5	98.5	98.2	97.8		41	62	72	111	114	116	117	141	180	269	285	162
7	*Glugea atherinae* (U15987)	**97.7**	97.7	97.7	97.3	96.9	96.6		30	78	120	123	125	125	109	146	238	256	174
8	*Glugea gasterostei* (KM977990)	**95.8**	95.8	95.8	95.4	95.0	94.9	97.4		98	143	146	148	148	121	122	214	276	195
9	*Glugea stephani* (AF056015)	**95.3**	95.3	95.3	95.0	94.7	94.1	93.5	91.9		134	137	139	139	162	201	309	221	181
10	*Glugea eda* (MK568064)	**92.3**	92.3	92.3	92.1	91.9	90.9	90.1	88.2	89		5	6	7	35	76	340	339	146
11	*Glugea serranus* (KU363832)	**92.0**	92.0	92.1	91.9	91.6	90.7	89.9	88.0	88.7	99.5		3	4	34	73	343	342	146
12	*Glugea nagelia* (KJ802012)	**91.8**	91.8	91.9	91.7	91.5	90.5	89.7	87.8	88.6	99.5	99.7		3	33	72	345	343	148
13	*Glugea arabica* (KT005391)	**91.8**	91.8	91.8	91.6	91.4	90.4	89.7	87.8	88.6	99.4	99.6	99.7		33	72	345	343	149
14	*Glugea epinephelusis* (AY090038)	**89.5**	89.5	89.5	89.3	89.1	88.4	90.9	89.8	86.6	97.1	97.2	97.2	97.2		47	318	329	176
15	*Glugea jazanensis* (KP262018)	**86.3**	86.3	86.4	86.1	85.9	85.3	87.8	89.6	83.4	93.7	94.0	94.0	94.0	96.0		274	367	212
16	*Glugea sardinellensis* (KU577431)*	**78.7**	*78.7*	*78.8*	*78.4*	*78.1*	*78.0*	*80.1*	*81.7*	*74.6*	*72.1*	*71.9*	*71.7*	*71.7*	*73.4*	*76.2*		485	391
17	*Glugea pagri* (JX852026)*	**77.6**	*77.6*	*77.6*	*77.3*	*77.0*	*76.7*	*78.6*	*76.9*	*81*	*72.2*	*71.9*	*71.8*	*71.8*	*72.5*	*69.4*	*59.6*		379
18	*Pleistophora typicalis* (AF044387)	**87.8**	87.8	87.9	87.4	87.4	86.9	85.9	84.2	85.3	88.1	88.1	88	87.9	85.7	82.8	68.3	69.3	

Table S3. Similarities and differences among representatives of the genera *Glugea* and *Pleistophora* for 16S rDNA sequences. Percent sequence identity percentage (below the diagonal) and pairwise nucleotide differences (above the diagonal) is based on alignment of **700** positions. Results between *Glugea* sp. 53 and the other microsporidia are shown in **bold**.

***Glugea* species**	**1**	**2**	**3**	**4**	**5**	**6**	**7**	**8**	**9**	**10**	**11**	**12**	**13**	**14**	**15**	**16**	**17**	**18**	**19**	**20**	**21**	**22**	**23**	**24**	**25**
**1. *Glugea* sp. 53 (MT072043)**		**0**	**0**	**0**	**0**	**1**	**1**	**1**	**1**	**2**	**2**	**2**	**3**	**5**	**6**	**7**	**12**	**25**	**64**	**67**	**67**	**68**	**68**	**68**	**98**
2. *Glugea* sp* GReina-2025a (KY882286)	**100**		0	0	0	1	1	1	1	2	2	2	3	5	6	7	12	25	64	67	67	68	68	68	98
3. *Glugea* sp.* ST1 (OR733697)	**100**	100		0	0	1	1	1	1	2	2	2	3	5	6	7	12	25	64	67	67	68	68	68	98
4. *Glugea thunni* (OM914139)	**100**	100	100		0	1	1	1	1	2	2	2	3	5	6	7	12	25	64	67	67	68	68	68	98
5. *Glugea hertwigi* (GQ203287)	**100**	100	100	100		1	1	1	1	2	2	2	3	5	6	7	12	25	64	67	67	68	68	68	98
6. *Glugea* sp. CSl-2020a clone 8 (MT680622)	**99.8**	99.8	99.8	99.8	99.8		2	2	2	3	3	3	4	6	7	8	13	26	65	68	68	69	69	69	99
7. *Glugea* sp. CSl-2020a clone 9 (MT680621)	**99.8**	99.8	99.8	99.8	99.8	99.6		2	2	3	3	3	4	6	7	8	13	26	63	66	66	67	67	67	98
8. *Glugea* sp.* ST2 (OR722585)	**99.8**	99.8	99.8	99.8	99.8	99.6	99.6		2	3	3	3	4	6	7	8	13	26	65	68	68	69	69	69	99
9. *Glugea gasterostei* (KM977990)	**99.8**	99.8	99.8	99.8	99.8	99.6	99.6	99.6		3	3	3	4	6	7	8	13	26	65	68	68	69	69	69	99
10. *Glugea* sp. CBG1 (KU885381)	**99.6**	99.6	99.6	99.6	99.6	99.5	99.5	99.5	99.5		4	4	4	7	8	7	14	25	65	68	68	69	69	69	99
11. *Glugea atherinae* (U15987)	**99.6**	99.6	99.6	99.6	99.6	99.5	99.5	99.5	99.5	99.3		4	5	7	8	9	14	27	66	69	69	70	70	70	100
12. *Glugea stephani* (AF056015)	**99.6**	99.6	99.6	99.6	99.6	99.5	99.5	99.5	99.5	99.3	99.3		5	7	8	9	14	27	66	69	69	70	70	70	100
13. *Glugea pagri* (JX852026)	**99.5**	99.5	99.5	99.5	99.5	99.3	99.3	99.3	99.3	99.3	99.2	99.2		8	9	9	15	27	65	68	68	69	69	69	99
14. *Glugea anomala* (AF044391)	**99.2**	99.2	99.2	99.2	99.2	99.0	99.0	99.0	99.0	98.9	98.9	98.9	98.7		11	12	17	30	69	72	72	73	73	73	102
15. *Glugea plecoglossi* (AB623035)	**99.0**	99.0	99.0	99.0	99.0	98.9	98.9	98.9	98.9	98.7	98.8	98.7	98.6	98.3		11	18	31	67	70	70	71	71	71	101
16. *Glugea* sp. CCG1 (KU885382)	**98.9**	98.9	98.9	98.9	98.9	98.7	98.7	98.7	98.7	98.9	98.6	98.6	98.6	98.1	98.3		19	30	68	71	71	72	72	72	102
17. *Glugea* sp. voucher 1_2 (PP864450)	**98.2**	98.2	98.2	98.2	98.2	98.0	98.0	98.0	98.0	97.9	97.9	97.9	97.7	97.4	97.3	97.1		37	76	79	79	80	80	80	110
18. *Glugea sardinellensis* (KU577431)	**96.2**	96.2	96.2	96.2	96.2	96.0	96.0	96.0	96.0	96.2	95.9	95.9	95.9	95.4	95.3	95.4	94.5		85	88	88	89	89	89	120
19. *Glugea eda* (MK568064)	**90.4**	90.4	90.4	90.4	90.4	90.2	90.5	90.2	90.2	90.2	90.1	90.1	90.2	89.6	90.0	89.8	88.8	87.3		5	5	5	5	5	95
20. *Glugea serranus* (KU363832)	**89.9**	89.9	89.9	89.9	89.9	89.8	90.1	89.8	89.8	89.8	89.7	89.6	89.8	89.2	89.5	89.3	88.3	86.8	99.2		2	4	2	2	95
21. *Glugea jazanensis* (KP262018)	**89.9**	89.9	89.9	89.9	89.9	89.8	90.1	89.8	89.8	89.8	89.7	89.6	89.8	89.2	89.5	89.3	88.3	86.8	99.2	99.6		4	2	2	94
22. *Glugea epinephelusis* (AY090038)	**89.8**	89.8	89.8	89.8	89.8	89.6	89.9	89.6	89.6	89.6	89.5	89.5	89.6	89.0	89.4	89.2	88.2	86.7	99.2	99.3	99.3		4	4	97
23. *Glugea nagelia* (KJ802012)	**89.8**	89.8	89.8	89.8	89.8	89.6	89.9	89.6	89.6	89.6	89.5	89.5	89.6	89.0	89.4	89.2	88.2	86.7	99.2	99.6	99.6	99.3		2	96
24. *Glugea arabica* (KT005391)	**89.8**	89.8	89.8	89.8	89.8	89.6	89.9	89.6	89.6	89.6	89.5	89.5	89.6	89.0	89.4	89.2	88.2	86.7	99.2	99.6	99.6	99.3	99.6		96
25. *Pleistophora typicalis* (AF044387)	**85.5**	85.5	85.5	85.5	85.5	85.4	85.5	85.4	85.4	85.4	85.3	85.2	85.4	84.9	85.1	84.9	84	82.3	86	86	86.1	85.7	85.8	85.8	

Table S4. Similarities and differences among representatives of the genus *Glugea* for 16S rDNA sequences. Percent sequence identity percentage (below the diagonal) and pairwise nucleotide differences (above the diagonal) is based on alignment of 1061 positions. Results between *Glugea* sp. 53 and the other microsporidia are shown in **bold**.

Glugea species		1	2	3	4	5	6	7	8	9	10	11	12	13
**1**	**Glugea sardina alacha 43-53 (Sardinella aurita)**		**0**	**0**	**1**	**2**	**2**	**2**	**3**	**3**	**3**	**5**	**9**	**10**
**2**	**KY882286.1 Glugea sp. GReina-2025a**	**100**		0	1	2	2	2	3	3	3	5	9	10
**3**	**OR733697 Glugea plecoglossi (ST1)**	**100**	100		1	2	2	2	3	3	3	5	9	10
**4**	**OM914139.1 Glugea thunni**	**99.9**	99.9	99.9		2	1	1	3	2	3	5	9	10
**5**	**OR722585.1 Glugea plecoglossi (ST2)**	**99.8**	99.8	99.8	99.8		3	3	3	4	3	5	9	10
**6**	**MT680621.1 Glugea sp. CSl-2020a clone 9**	**99.8**	99.8	99.8	99.9	99.7		2	4	3	4	6	10	11
**7**	**KM977990 Glugea gasterostei**	**99.8**	99.8	99.8	99.9	99.7	99.8		4	3	4	6	10	11
**8**	**MT680622.1 Glugea sp. CSl-2020a clone 8**	**99.7**	99.7	99.7	99.7	99.7	99.6	99.6		5	4	6	10	11
**9**	**U15987 Glugea atherinae**	**99.7**	99.7	99.7	99.8	99.6	99.7	99.7	99.5		5	7	11	12
**10**	**AF056015 Glugea stephani**	**99.7**	99.7	99.7	99.7	99.7	99.6	99.6	99.6	99.5		6	10	11
**11**	**GQ203287 Glugea hertwigi**	**99.5**	99.5	99.5	99.5	99.5	99.4	99.4	99.4	99.3	99.4		12	13
**12**	**AB623035 Glugea plecoglossi**	**99.1**	99.1	99.1	99.1	99.1	99.0	99.0	99.0	98.9	99.0	98.8		17
**13**	**AF044391 Glugea anomala**	**99.0**	99.0	99.0	99.0	99.0	98.9	98.9	98.9	98.8	98.9	98.7	98.3	

Table S5. Similarities and differences among representatives of the genera *Glugea* and *Pleistophora* for Internal transcribe region and partial 23S rDNA sequences. Percent sequence identity percentage (below the diagonal) and pairwise nucleotide differences (above the diagonal) is based on alignment of 417 positions. Results between *Glugea* sp. 53 and the other microsporidia are shown in **bold**.

***Glugea* species**	**1**	**2**	**3**	**4**	**5**	**6**	**7**	**8**	**9**	**10**	**11**	**12**	**13**	**14**
1. *Glugea* sp. 53 (MT072043)		**0**	**0**	**1**	**4**	**7**	**8**	**10**	**11**	**15**	**78**	**80**	**82**	**110**
2. *Glugea* sp. GReina-2025a (KY882286)	**100**		0	1	4	7	8	10	11	15	78	80	82	110
3. *Glugea thunni* (OM914139)	**100**	100		1	4	7	8	10	11	15	78	80	82	110
4. *Glugea* sp. voucher 1_2 (PP864450)	**99.7**	99.7	99.7		5	8	8	10	12	16	78	80	82	110
5. *Glugea* sp. GS1 (AJ295325)	**99.0**	99.0	99.0	98.8		7	7	10	11	15	78	80	82	109
6. *Glugea* sp. CBG1 freshwater sculpins (KU885381)	**98.3**	98.3	98.3	98.0	98.3		9	15	8	18	80	82	84	113
7. *Glugea plecoglossi* (AJ295326)	**98.0**	98.0	98.0	98.0	98.3	97.8		15	13	19	78	80	82	110
8. *Glugea hertwigi* (GQ203287)	**97.6**	97.6	97.6	97.6	97.6	96.4	96.4		19	21	79	81	83	110
9. *Glugea* sp. CCG1 freshwater sculpins (KU885382)	**97.3**	97.3	97.3	97.1	97.3	98.0	96.8	95.4		21	84	86	88	117
10. *Glugea anomala* (AF044391)	**96.4**	96.4	96.4	96.2	96.4	95.7	95.4	95.0	95.0		89	91	93	123
11. *Glugea serranus* (KU363832)	**81.2**	81.2	81.2	81.2	81.2	80.8	81.2	81.1	79.8	78.9		4	8	95
12. *Glugea nagelia* (KJ802012)	**80.8**	80.8	80.8	80.8	80.8	80.3	80.8	80.6	79.3	78.4	99.0		10	94
13. Glugea arabica (KT005391)	**80.3**	80.3	80.3	80.3	80.3	79.8	80.3	80.1	78.8	77.9	98.0	97.5		101
14. *Pleistophora typicalis* (AF044387)	**73.9**	73.9	73.9	73.9	74.2	73.2	73.9	74.0	72.3	71.2	77.5	77.7	76.1	

Table S6. Similarities and differences among representatives of the genera *Glugea* and *Pleistophora* for partial 16S rDNA, Internal transcribe region and partial 23S rDNA sequences. Percent sequence identity percentage (below the diagonal) and pairwise nucleotide differences (above the diagonal) is based on alignment of 1686 positions. Results between *Glugea* sp. 53 and the other microsporidia are shown in **bold**.

	**Seq->**	**1**	**2**	**3**	**4**	**5**	**6**	**7**	**8**	**9**	**10**	**11**
**1**	**Glugea sardina alacha 43-53 (Sardinella aurita)**		**0**	**1**	**14**	**22**	**18**	**31**	**189**	**191**	**194**	**258**
**2**	**KY882286.1 Glugea sp.* GReina-2025a**	**100**		1	14	22	18	31	189	191	194	258
**3**	**OM914139.1 Glugea thunni**	**99.9**	99.9		14	22	18	31	188	190	193	257
**4**	**PP864450.1 Glugea sp. voucher 1_2**	**99.1**	99.1	99.1		34	30	43	202	204	207	271
**5**	**KU885382.1 Glugea sp. CCG1**	**98.6**	98.6	98.6	97.9		36	46	201	203	206	269
**6**	**GQ203287 Glugea hertwigi**	**98.8**	98.8	98.8	98.1	97.7		43	195	197	200	265
**7**	**AF044391 Glugea anomala**	**98.1**	98.1	98.1	97.3	97.1	97.3		214	216	219	282
**8**	**KU363832 Glugea serranus**	**88.4**	88.4	88.5	87.7	87.7	88	86.9		7	11	236
**9**	**KJ802012 Glugea nagelia**	**88.3**	88.3	88.3	87.6	87.5	87.9	86.8	99.5		12	238
**10**	**KT005391 Glugea arabica**	**88.1**	88.1	88.1	87.4	87.4	87.7	86.6	99.3	99.2		245
**11**	**AF044387 Pleistophora typicalis**	**84.3**	84.3	84.4	83.7	83.7	83.9	83	85.7	85.5	85.1	
